# 1-year weight change after diabetes diagnosis and long-term incidence and sustainability of remission of type 2 diabetes in real-world settings in Hong Kong: An observational cohort study

**DOI:** 10.1371/journal.pmed.1004327

**Published:** 2024-01-23

**Authors:** Hongjiang Wu, Aimin Yang, Eric S. H. Lau, Xinge Zhang, Baoqi Fan, Ronald C. W. Ma, Alice P. S. Kong, Elaine Chow, Wing-Yee So, Juliana C. N. Chan, Andrea O. Y. Luk

**Affiliations:** 1 Department of Medicine and Therapeutics, The Chinese University of Hong Kong, Hong Kong, China; 2 Hong Kong Institute of Diabetes and Obesity, The Chinese University of Hong Kong, Hong Kong, China; 3 Li Ka Shing Institute of Health Sciences, The Chinese University of Hong Kong, Hong Kong, China; 4 Hong Kong Hospital Authority, Hong Kong Special Administrative Region, China

## Abstract

**Background:**

Clinical trials have demonstrated that remission of type 2 diabetes can be achieved following sustained weight loss. However, the feasibility of achieving diabetes remission through weight management in real-world settings remains unclear. In this study, we aimed to examine the association of weight change at 1 year after diabetes diagnosis with long-term incidence and sustainability of type 2 diabetes remission in real-world settings in Hong Kong.

**Methods and findings:**

This was a population-based observational cohort study. The territory-wide Risk Assessment and Management Programme for Diabetes Mellitus (RAMP-DM) provides regular comprehensive assessments of metabolic control and complication screening for people with diabetes in Hong Kong. We included 37,326 people with newly diagnosed type 2 diabetes who were enrolled in the RAMP-DM between 2000 and 2017, followed until 2019. Diabetes remission was defined as 2 consecutive HbA1c <6.5% measurements at least 6 months apart in the absence of glucose-lowering drugs (GLDs) and with no record of GLDs at least 3 months before these measurements. During a median follow-up of 7.9 years, 6.1% (2,279) of people achieved diabetes remission, with an incidence rate of 7.8 (95% CI: 7.5, 8.1) per 1,000 person-years. After adjusting for age at diabetes diagnosis, sex, assessment year, body mass index, other metabolic indices, smoking, alcohol drinking, and medication use, the hazard ratio (HR) for diabetes remission was 3.28 (95% CI: 2.75, 3.92; *p* < 0.001) for people with ≥10% weight loss within 1 year of diagnosis, 2.29 (95% CI: 2.03, 2.59; *p* < 0.001) for those with 5% to 9.9% weight loss, and 1.34 (95% CI: 1.22, 1.47; *p* < 0.001) for those with 0% to 4.9% weight loss compared to people with weight gain. During a median follow-up of 3.1 years, 67.2% (1,531) of people who had achieved diabetes remission returned to hyperglycaemia, with an incidence rate of 184.8 (95% CI: 175.5, 194.0) per 1,000 person-years. The adjusted HR for returning to hyperglycaemia was 0.52 (95% CI: 0.41, 0.65; *p* < 0.001) for people with ≥10% weight loss, 0.78 (95% CI: 0.68, 0.92; *p* = 0.002) for those with 5% to 9.9% weight loss, and 0.90 (95% CI: 0.80, 1.01; *p* = 0.073) for those with 0% to 4.9% weight loss compared to people with weight gain. Diabetes remission was associated with a 31% (HR: 0.69, 95% CI: 0.52, 0.93; *p* = 0.014) decreased risk of all-cause mortality. The main limitation of the study is that the reliability of HbA1c used to define diabetes remission can be affected by other medical conditions. Furthermore, we did not have data on bariatric surgery.

**Conclusions:**

In this study, greater weight loss within the first year of diabetes diagnosis was associated with an increased likelihood of achieving diabetes remission and a decreased risk of returning to hyperglycaemia among those who had achieved diabetes remission. However, both the incidence of diabetes remission and the probability of its long-term sustainability were low with conventional management in real-world settings, in an era when the importance of weight loss was not fully appreciated. Our study provides evidence for policymakers to design and implement early weight management interventions and diabetes remission initiatives.

## Abstract 2

In this population based cohort study of >37,000 people in Hong Kong, Hongjiang Wu and colleagues examine 1-year weight change after diabetes diagnosis with long-term incidence and sustainability of diabetes remission.

## Author summary

**Why was this study done?:**

- Remission of type 2 diabetes is defined as a return to normal blood glucose levels without the need for pharmacotherapy.
- Recent clinical trials have demonstrated that remission of type 2 diabetes can be achieved following sustained weight loss through bariatric surgery or lifestyle interventions.
- However, the feasibility of achieving diabetes remission through weight management and its long-term sustainability in real-world settings remain unclear.

**What did the researchers do and find?:**

- Using data from 37,326 people with newly diagnosed type 2 diabetes from a diabetes complication screening program in Hong Kong, we examined the associations of 1-year weight change (%) after diabetes diagnosis with long-term incidence and sustainability of diabetes remission.
- We found only 6% of people achieved diabetes remission during a median follow-up of 8 years. Among people who achieved diabetes remission, 67% of them returned to hyperglycaemia during a median follow-up of 3 years.
- Compared to people who had weight gain, those who had a greater 1-year weight loss (%) after diabetes diagnosis were more likely to achieve diabetes remission and had a decreased risk of returning to hyperglycaemia.

**What do these findings mean?:**

- Remission of type 2 diabetes is achievable in real-world settings, but both the incidence of diabetes remission and the probability of its long-term sustainability are low with conventional management.
- Implementation of early weight management interventions should be considered as diabetes remission initiatives.
- A limitation of the study is that the reliability of HbA1c as a measure for diabetes remission can be influenced by other medical conditions.

## Introduction

Type 2 diabetes is characterised by persistent hyperglycaemia and has traditionally been considered an irreversible condition that requires lifelong drug treatment for glucose control. However, recent clinical trials have demonstrated that remission of type 2 diabetes, defined as a return to normal blood glucose levels without the need for pharmacotherapy, can be achieved following sustained weight loss through bariatric surgery [1,2] or lifestyle interventions [3,4] in those who were overweight or obese. The STAMPEDE trial showed that around one-third of obese people with type 2 diabetes achieved a haemoglobin A1C (HbA1c) level of less than 6% without glucose-lowering drugs (GLDs) 1 year after bariatric surgery [1]. In the Diabetes Remission Clinical Trial (DiRECT), approximately half of the participants with type 2 diabetes achieved a HbA1c level of less than 6.5% without GLDs after losing an average of 10 kg body weight through a primary care–led weight management intervention over a 12-month period [3].

However, our understanding of diabetes remission remains limited, largely because the majority of existing evidence comes from clinical trials. The strictly controlled environments and selective participants in clinical trials have inherently limited the generalisability of their findings to broader populations. Evidence from more diverse populations is needed to better understand the feasibility of achieving diabetes remission through weight management in real-world settings to inform clinical practice. More importantly, given the short follow-up time of clinical trials, the long-term incidence, sustainability, and benefits of diabetes remission are largely unknown.

Weight control is a key component in the management of type 2 diabetes. Effective management of body weight during the early stage of diabetes disease trajectory can prevent diabetes-related complications and improve long-term outcomes [5]. The first year after diabetes diagnosis represents a critical period for early intervention and reflects the initial response to lifestyle changes and treatment [6,7]. In this study, we aimed to use real-world data from Hong Kong Chinese to examine (1) the associations between 1-year weight change with conventional management after diabetes diagnosis and the long-term incidence and sustainability of remission of type 2 diabetes; and (2) the association between diabetes remission and all-cause and cause-specific mortality.

## Methods

### Data source and study setting

Details on the Hong Kong Hospital Authority (HA) Electronic Medical Record (EMR) system and the Risk Assessment and Management Programme for Diabetes Mellitus (RAMP-DM) have been reported [8–12]. Briefly, the Hong Kong HA is a statutory body established in 1990 that governs all public hospitals and the majority of specialist and general outpatient clinics. Due to the highly subsidised public healthcare system, the HA provides about 90% of total health services in Hong Kong. In 2000, the HA implemented a territory-wide RAMP-DM in 18 hospital-based diabetes centres to provide regular comprehensive risk assessment and complication screening to people with diabetes referred from outpatient clinics. In 2009, the HA expanded the RAMP-DM from hospital-based diabetes centres to all general outpatient clinics in primary care settings. All people with diabetes were eligible to participate in the RAMP-DM with no specific criteria, and approximately 60% of the Hong Kong population with diabetes have been enrolled in this program. We planned the study in December 2022, conducted the analyses between January and July 2023, and performed additional analyses in October 2023 in response to suggestions from journal reviewers. This study is reported as per the Strengthening the Reporting of Observational Studies in Epidemiology (STROBE) guideline (S1 STROBE Checklist).

### Study population

**Fig 1:**
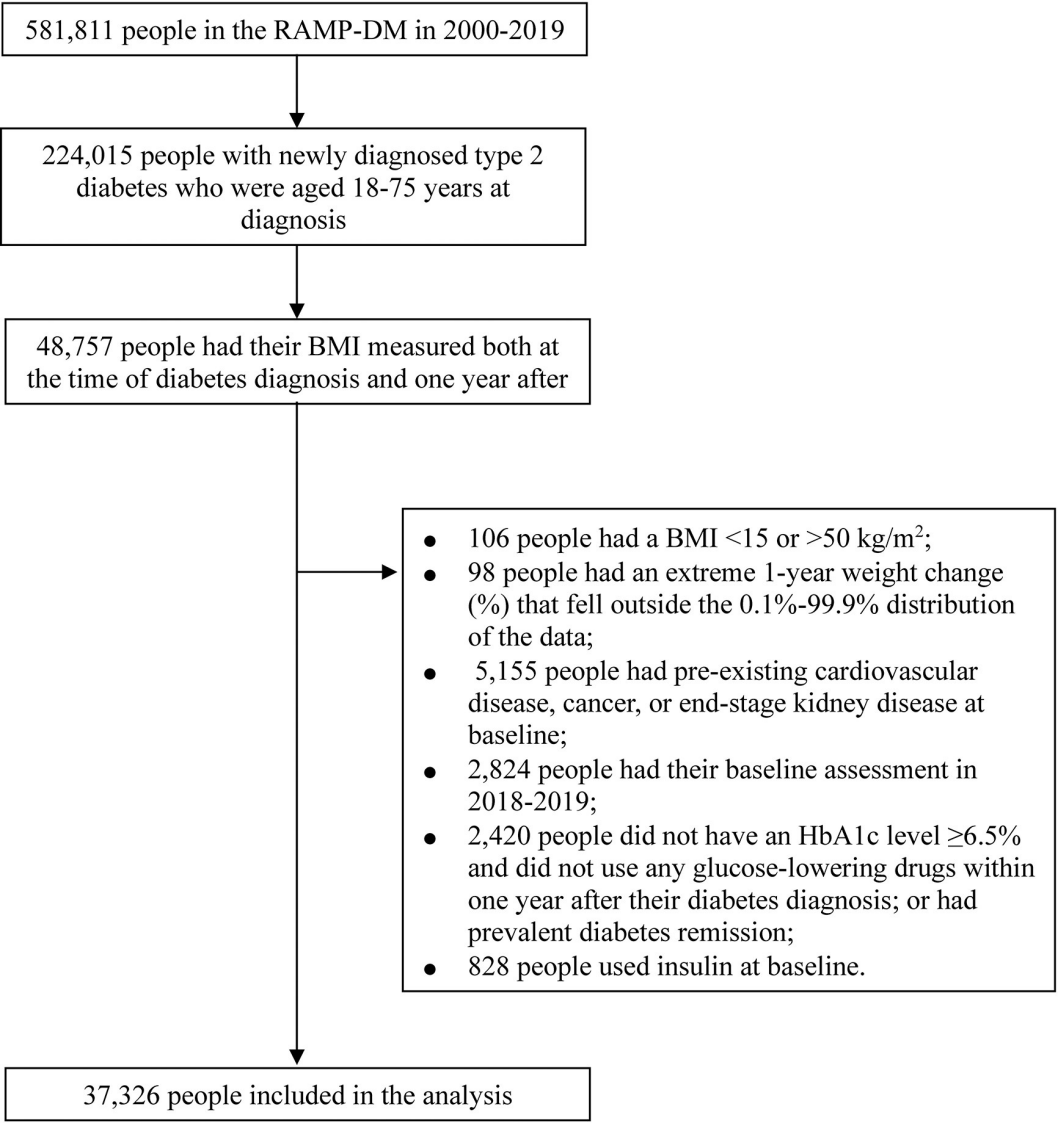
Flowchart of the selection of participants in the Risk Assessment and Management Programme for Diabetes Mellitus (RAMP-DM).

We included people with newly diagnosed type 2 diabetes (self-reported diabetes duration ≤1 year) who underwent their first RAMP-DM assessment between 1 January 2000 and 31 December 2017. To reduce the potential influence of age-related weight changes, we excluded people who were younger than 18 years (weight would increase as part of pubertal development) or older than 75 years (weight could decrease due to age-related loss of muscle mass) at diabetes diagnosis. Furthermore, we excluded people who had an extreme BMI (<15 or >50 kg/m^2^), had potentially unreliable values on 1-year weight change (%) falling outside the 0.1% to 99.9% range of the data distribution, or had missing data on 1-year weight change. To reduce the probability that weight changes were caused by severe illness, we excluded people with preexisting cardiovascular disease, cancer, or end-stage renal disease. Additionally, we excluded people who did not use any GLDs but with no record of an HbA1c ≥6.5% (48 mmol/mol) within 1 year after diabetes diagnosis or those with prevalent diabetes remission. We also excluded people who used insulin at baseline as these individuals might have type 1 diabetes. Finally, a total number of 37,326 people were included in the analysis (**Fig 1**).

### Assessment of 1-year weight change

The exposure variable was the percentage of 1-year weight change (%) after diabetes diagnosis. We used the body weight measured at the first RAMP-DM assessment at diabetes diagnosis as the baseline weight and the body weight at the follow-up RAMP-DM assessment conducted closest (±6 months) to 1 year after the baseline as the 1-year weight.

### Outcome measures

The primary outcome was incident remission of type 2 diabetes. We defined diabetes remission based on the 2021 International Consensus report as 2 or more consecutive HbA1c <6.5% (48 mmol/mol) measurements at a time interval of at least 6 months in the absence of GLDs between these measurements and with no record of GLDs at least 3 months before the first HbA1c <6.5% [13,14]. The date of the first HbA1c <6.5% was used as the date of incident diabetes remission. The secondary outcomes included the following: (1) incident return to hyperglycaemia, defined as an HbA1c ≥6.5% or use of GLDs among people who had achieved diabetes remission; and (2) all-cause and cause-specific mortality among people with and without diabetes remission. Cause-specific mortality included mortality due to cancer (ICD-10: C00-C97), cardiovascular disease (ICD-10: I00-I99, F01 and G45), and pneumonia (ICD-10: J12-J18), which were the 3 most common causes of death in type 2 diabetes in Hong Kong [11]. Routinely collected data on HbA1c, GLD prescriptions, and mortality were obtained from the HA EMR database.

### Definition of follow-up period

In the analysis of diabetes remission, people were followed from their baseline RAMP-DM assessment to the date of incident diabetes remission, death, or 31 December 2019, whichever came first. In the analysis of return to hyperglycaemia, people were followed from the date of diabetes remission to the date of incident return to hyperglycaemia, death, or 31 December 2019, whichever came first. In the analysis of mortality, to reduce immortal time bias [15], people with diabetes remission were followed up from the date of diabetes remission, while those without diabetes remission were followed up from their baseline RAMP-DM assessment to death or 31 December 2019, whichever came first.

### Statistical analysis

We categorised the study population based on their 1-year weight change (%) into 4 groups: (1) weight loss of ≥10%; (2) weight loss of 5% to 9.9%; (3) weight loss of 0% to 4.9%; and (4) weight gain of >0%. The decision to categorise 1-year weight change (%) and the selection of cutoffs were made to align with previous publications for comparison purposes [3,4,14,16].

We fitted multivariable Cox proportional-hazards regression models to estimate the hazard ratios (HRs) and 95% confidence intervals (CIs) for the associations of 1-year weight change (%) categories after diabetes diagnosis with incident diabetes remission and incident return to hyperglycaemia, as well as for the association of diabetes remission with all-cause and cause-specific mortality. Subgroup analyses were performed according to the key characteristics of the study population. We checked the Cox proportional hazards (PHs) assumption using Schoenfeld residuals. We found that the assumption was violated for the association between 1-year weight change (%) categories and incident return to hyperglycaemia but not for other models. Thus, the HRs were interpreted as an average effect during the entire follow-up period [17]. To explore the possible nonlinear relationship between 1-year weight change (%) as a continuous variable and diabetes remission, a restricted cubic spline term with 5 knots placed at 2.5%, 25%, 50%, 75%, and 97.5% through the data distribution was included in the model. The number of knots in the spline was selected based on the Bayesian information criterion. We managed missing data using the multiple imputation by chained equations (MICE) method and assuming that data were missing at random (**S1 Table**). We generated 15 imputed datasets based on the greatest percentage of incomplete cases in the dataset, and we pooled the results using the Rubin’s rule [18].

### Additional and sensitivity analyses

For comparison purposes, we examined the associations of 1-year change (%) in waist circumference with incident diabetes remission and incident return to hyperglycaemia. Waist circumference is a measure of central obesity and is considered an important risk factor for metabolic disease in Asians. In addition, the definition of diabetes remission based on 2 HbA1c measurements taken at least 6 months apart could potentially introduce immortal time bias as people who did not survive to receive the second HbA1c measurement were inherently classified as not having achieved diabetes remission. Therefore, we performed a sensitivity analysis excluding people who died within 6 months after achieving their first HbA1c measurement <6.5% to examine the association between diabetes remission and all-cause mortality. Moreover, given the high prevalence of cardiovascular disease in people with diabetes, we further examined the association between 1-year weight change (%) and incident diabetes remission by including those (*n* = 2,956) with preexisting cardiovascular disease to enhance the generalisability of the results. We also compared the characteristics of people included in the analysis with those excluded due to missing data on weight change but who still met other inclusion criteria.

All analyses were performed using R software, version 4.0.3 (R Foundation for Statistical Computing, Vienna, Austria). This study was approved by the Chinese University of Hong Kong-New Territories East Cluster Clinical Research Ethics Committee (CREC Ref. No. 2020.032).

## Results

Among the 37,326 people included in the analysis, 50.5% (*n* = 18,832) were men. At baseline, the mean age was 56.6 (standard deviation [SD]: 9.9) years, the mean BMI was 26.4 (SD: 4.2) kg/m^2^, the mean HbA1c was 7.7% (SD: 1.8%), and 65.0% were using GLDs (**Table 1**). On average, people experienced a 0.2% (SD: 5.2%) reduction in body weight 1 year after diabetes diagnosis (**Table 1**). Overall, 2.8% of people had a 1-year weight loss of ≥10%, 10.4% had a weight loss of 5% to 9.9%, 40.2% had a weight loss of 0% to 4.9%, and 46.6% had weight gain. People who had a greater 1-year weight loss were more likely to be women, had higher blood pressure and lipid levels, were less likely to be current smokers and alcohol users, and were less likely to use GLDs at baseline. They had higher levels of BMI, waist circumference, and HbA1c at baseline, but lower levels for these measurements 1 year after baseline. There was no clear trend in age at diabetes diagnosis and proportion of people using blood pressure–lowering drugs or lipid-lowering drugs at baseline across the weight change groups.

**Table 1:** Baseline characteristics and selected 1-year measures of the study population according to 1-year weight change (%) after diabetes diagnosis.

Characteristics	1-year weight change (%) group				Overall
	≥10% weight loss	5% to 9.9% weight loss	0% to 4.9% weight loss	>0% weight gain	
Number (%)	1,059 (2.8)	3,864 (10.4)	15,016 (40.2)	17,387 (46.6)	37,326 (100)
Age at diabetes diagnosis (years)	56.4 (10.6)	57.3 (9.8)	57.2 (9.7)	56.0 (10.0)	56.6 (9.9)
Male sex	440 (41.5)	1,717 (44.4)	7,326 (48.8)	9,349 (53.8)	18,832 (50.5)
Assessment year					
2000–2009	351 (33.1)	1,349 (34.9)	5,159 (34.4)	5,898 (33.9)	12,757 (34.2)
2010–2013	438 (41.4)	1,465 (37.9)	5,561 (37.0)	6,285 (36.1)	13,749 (36.8)
2014–2017	270 (25.5)	1,050 (27.2)	4,296 (28.6)	5,204 (29.9)	10,820 (29.0)
BMI category					
<24 kg/m^2^	253 (23.9)	1,021 (26.4)	3,938 (26.2)	5,906 (34.0)	11,118 (29.8)
24–27.9 kg/m^2^	407 (38.4)	1,572 (40.7)	6,142 (40.9)	6,902 (39.7)	15,023 (40.2)
≥28 kg/m^2^	399 (37.7)	1,271 (32.9)	4,936 (32.9)	4,579 (26.3)	11,185 (30.0)
BMI (kg/m^2^) at baseline	27.4 (4.7)	26.7 (4.1)	26.7 (4.1)	25.9 (4.2)	26.4 (4.2)
BMI (kg/m^2^) at 1 year	23.6 (3.9)	24.8 (3.9)	26.2 (4.0)	26.9 (4.3)	26.3 (4.2)
Weight (kg) at baseline	69.0 (14.6)	67.6 (12.7)	68.3 (12.7)	67.0 (13.0)	67.6 (12.9)
Weight (kg) at 1 year	59.5 (12.3)	63.0 (11.9)	66.9 (12.5)	69.5 (13.4)	67.5 (13.1)
1-year absolute weight change (kg)	−9.6 (3.8)	−4.6 (1.3)	−1.4 (1.0)	2.5 (2.4)	−0.2 (3.5)
1-year weight change (%)	−13.7 (3.7)	−6.9 (1.3)	−2.1 (1.4)	3.8 (3.7)	−0.2 (5.2)
Central obesity (%)	721 (75.7)	2,456 (71.8)	9,532 (71.8)	9,817 (63.9)	22,526 (68.3)
Waist circumference (cm) at baseline					
Men	94.1 (11.4)	92.4 (10.0)	92.3 (9.8)	90.6 (10.4)	91.5 (10.2)
Women	88.6 (10.0)	88.0 (10.1)	88.7 (10.2)	87.2 (10.5)	87.9 (10.3)
Waist circumference (cm) at 1 year					
Men	86.0 (10.6)	88.1 (9.7)	91.1 (9.6)	92.8 (10.1)	91.6 (10.0)
Women	81.6 (9.6)	84.4 (9.7)	87.6 (10.0)	89.1 (10.4)	87.7 (10.3)
1-year waist circumference change (%)					
Men	−8.0 (6.7)	−4.6 (5.0)	−1.2 (4.8)	2.6 (5.5)	0.2 (5.9)
Women	−7.4 (6.8)	−4.0 (6.5)	−1.0 (6.0)	2.4 (6.5)	−0.1 (6.8)
HbA1c at baseline					
%	7.7 (1.7)	7.6 (1.5)	7.5 (1.5)	7.8 (2.1)	7.7 (1.8)
mmol/mol	61.3 (18.7)	59.8 (16.6)	58.2 (16.3)	62.1 (23.0)	60.3 (19.9)
HbA1c at 1 year					
%	6.4 (1.1)	6.6 (1.0)	6.8 (0.9)	7.0 (1.0)	6.9 (1.0)
mmol/mol	46.1 (12.4)	48.6 (11.4)	51.4 (10.3)	53.2 (11.2)	51.8 (11.0)
1-year HbA1c change					
%	−1.4 (1.8)	−1.0 (1.6)	−0.6 (1.5)	−0.8 (2.0)	−0.8 (1.8)
mmol/mol	−15.0 (20.1)	−11.3 (17.4)	−6.8 (15.9)	−9.0 (22.1)	−8.6 (19.4)
Blood pressure (mm Hg)					
SBP	135.1 (17.9)	135.3 (18.0)	134.6 (17.0)	132.8 (17.7)	133.8 (17.5)
DBP	77.7 (10.4)	78.0 (10.3)	78.2 (10.0)	77.8 (10.3)	78.0 (10.2)
Total cholesterol (mmol/L)	5.1 (1.1)	5.1 (1.1)	5.0 (1.0)	4.9 (1.0)	5.0 (1.0)
LDL-C (mmol/L)	3.1 (0.9)	3.1 (0.9)	3.0 (0.9)	2.9 (0.9)	3.0 (0.9)
HDL-C (mmol/L)	1.3 (0.3)	1.3 (0.3)	1.2 (0.3)	1.2 (0.3)	1.3 (0.3)
Triglycerides (mmol/L)	1.4 (0.9, 1.9)	1.4 (1.0, 2.0)	1.5 (1.0, 2.1)	1.4 (1.0, 2.0)	1.4 (1.0, 2.0)
eGFR (mL/min/1.73 m^2^)	89.4 (17.3)	89.6 (16.2)	89.5 (15.9)	91.2 (16.4)	90.3 (16.2)
Smoking status					
Current	120 (12.1)	441 (12.5)	1,924 (13.9)	2,579 (16.0)	5,064 (14.7)
Former	108 (10.9)	446 (12.6)	1,936 (14.0)	2,458 (15.3)	4,948 (14.3)
Never	763 (77.0)	2,652 (74.9)	9,994 (72.1)	11,073 (68.7)	24,482 (71.0)
Alcohol drinking status					
Current	182 (18.5)	739 (21.0)	3,171 (23.2)	3,741 (23.6)	7,833 (23.0)
Former	69 (7.0)	244 (6.9)	992 (7.2)	1,380 (8.7)	2,685 (7.9)
Never	733 (74.5)	2,529 (72.0)	9,529 (69.6)	10,731 (67.7)	23,522 (69.1)
Oral glucose-lowering drugs (yes)					
Any	609 (57.5)	2,287 (59.2)	9,360 (62.3)	11,998 (69.0)	24,254 (65.0)
Metformin	548 (51.7)	2,049 (53.0)	8,151 (54.3)	10,151 (58.4)	20,899 (56.0)
Sulfonylureas	161 (15.2)	584 (15.1)	3,071 (20.5)	5,074 (29.2)	8,890 (23.8)
Others	3 (0.3)	4 (0.1)	20 (0.1)	62 (0.4)	89 (0.2)
Blood pressure–lowering drugs (yes)	564 (53.3)	2,122 (54.9)	8,639 (57.5)	8,724 (50.2)	20,049 (53.7)
Lipid-lowering drugs (yes)	167 (15.8)	693 (17.9)	3,026 (20.2)	3,339 (19.2)	7,225 (19.4)
Number (%) of people achieved diabetes remission	152 (14.4)	384 (9.9)	969 (6.5)	774 (4.5)	2,279 (6.1)

Data are mean (standard deviation), median (interquartile range), or n (%) as appropriate. Summary statistics are reported based on the complete data for each variable. Proportion of missing data for each variable is shown in the S1 Table. All *p*-values for the comparisons across weight change groups are less than 0.05. Central obesity is defined as waist circumference ≥90 cm in men and waist circumference ≥80 cm in women. Abbreviations: BMI, body mass index; DBP, diastolic blood pressure; eGFR, estimated glomerular filtration rate, HbA1c, haemoglobin A1c; HDL-C, high-density lipoprotein cholesterol; LDL-C, low-density lipoprotein; SBP, systolic blood pressure.

### Weight change and incident diabetes remission

**Fig 2:**
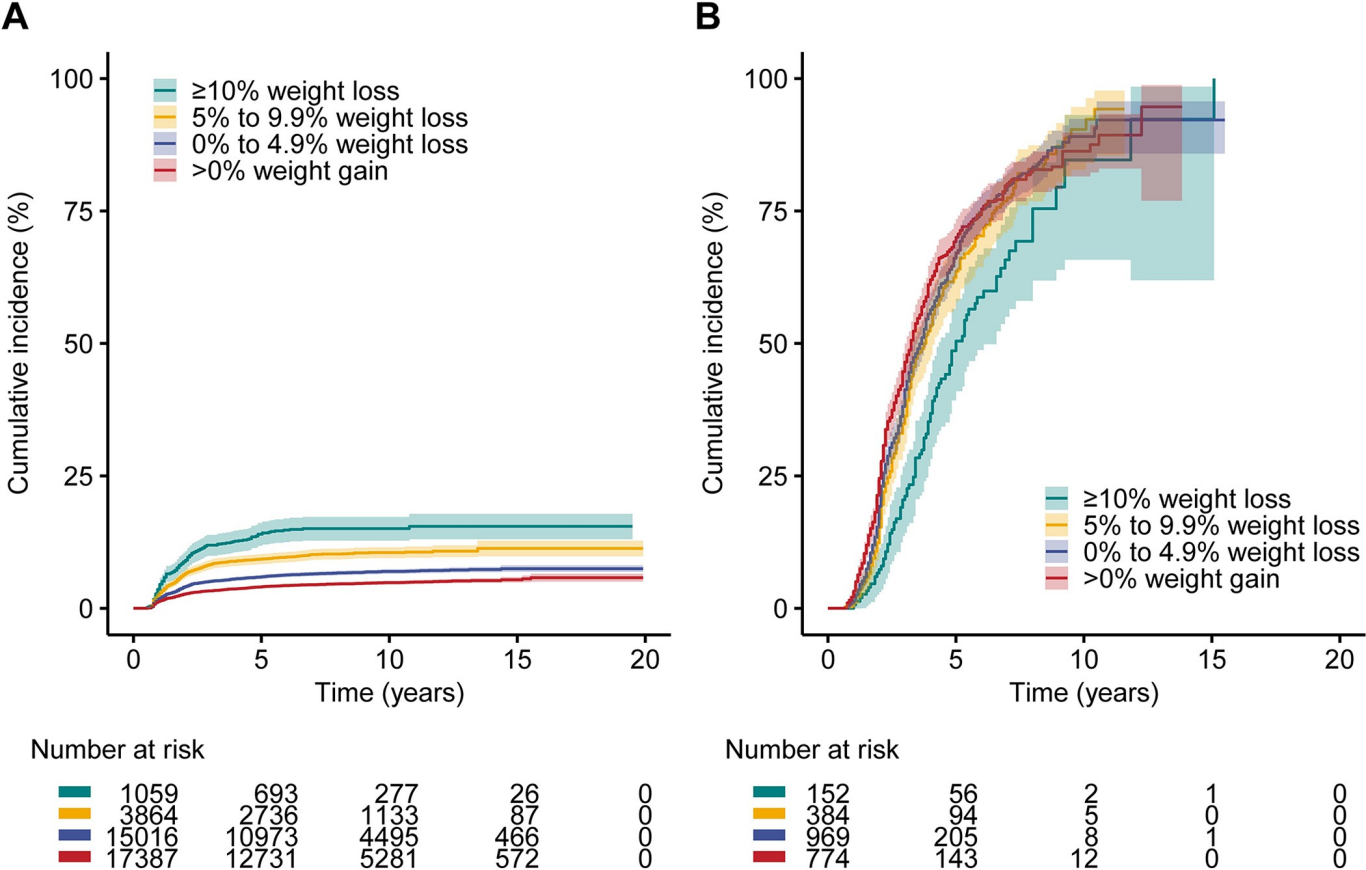
Kaplan–Meier plot of cumulative incidence of remission of type 2 diabetes (A) and cumulative incidence of return to hyperglycaemia among people with diabetes remission (B) according to 1-year weight change (%) after diabetes diagnosis.

**Fig 3:**
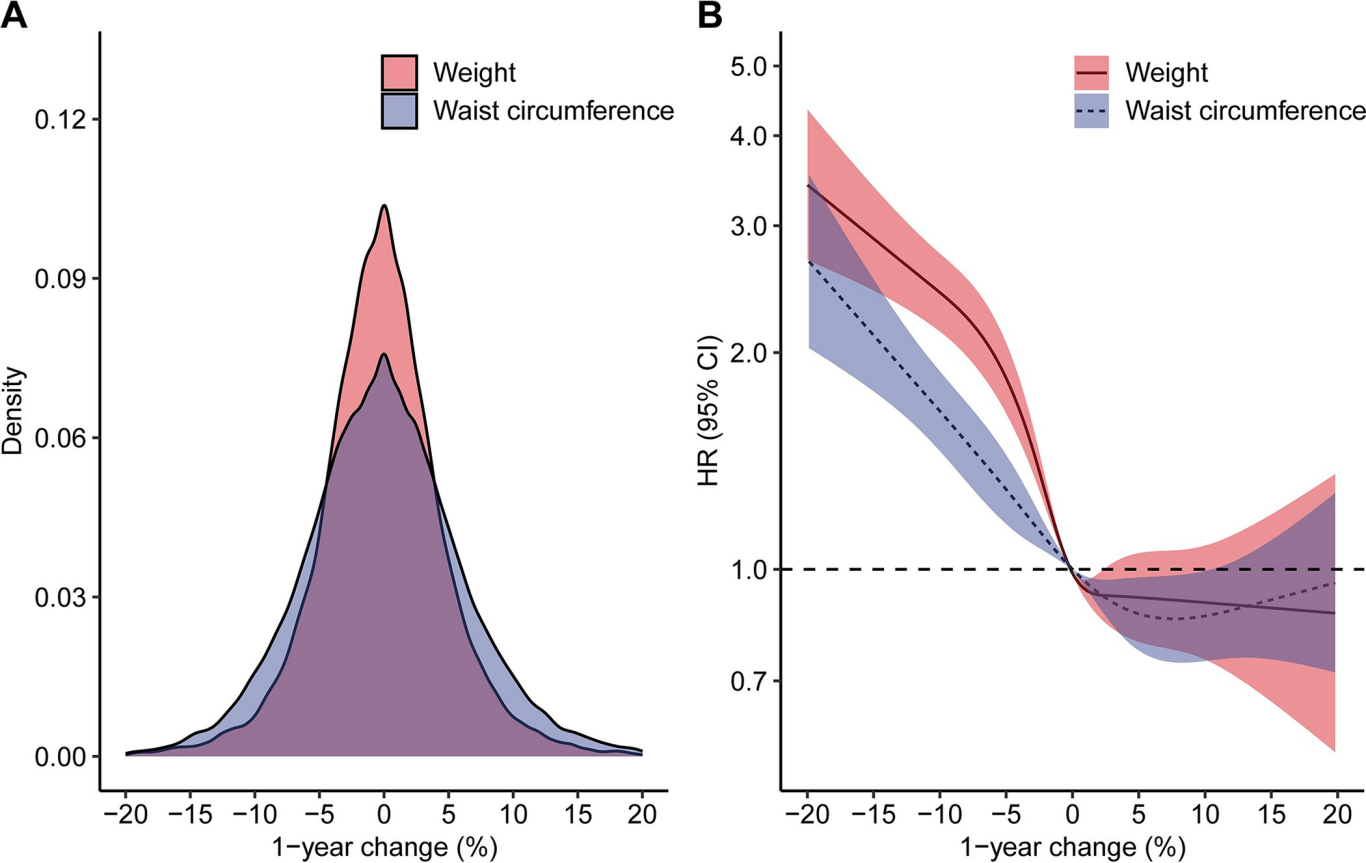
Density plots (A) and restricted cubic splines (B) of the associations of 1-year change (%) in weight and waist circumference after diabetes diagnosis with incident remission of type 2 diabetes. Density plots show the distribution of 1-year change (%) in weight and waist circumference. A negative value indicates weight and waist circumference loss. In the restricted cubic splines, fully adjusted Cox regression models were fitted with 5 knots at 2.5%, 25%, 50%, 75%, and 97.5% through the data distribution. The reference value for each hazard ratio (HR) of 1.0 was set as 0% change. Solid/dashed lines are HR estimates, with shaded areas showing 95% confidence intervals. The y-axis is natural log-transformed.

**Fig 4:**
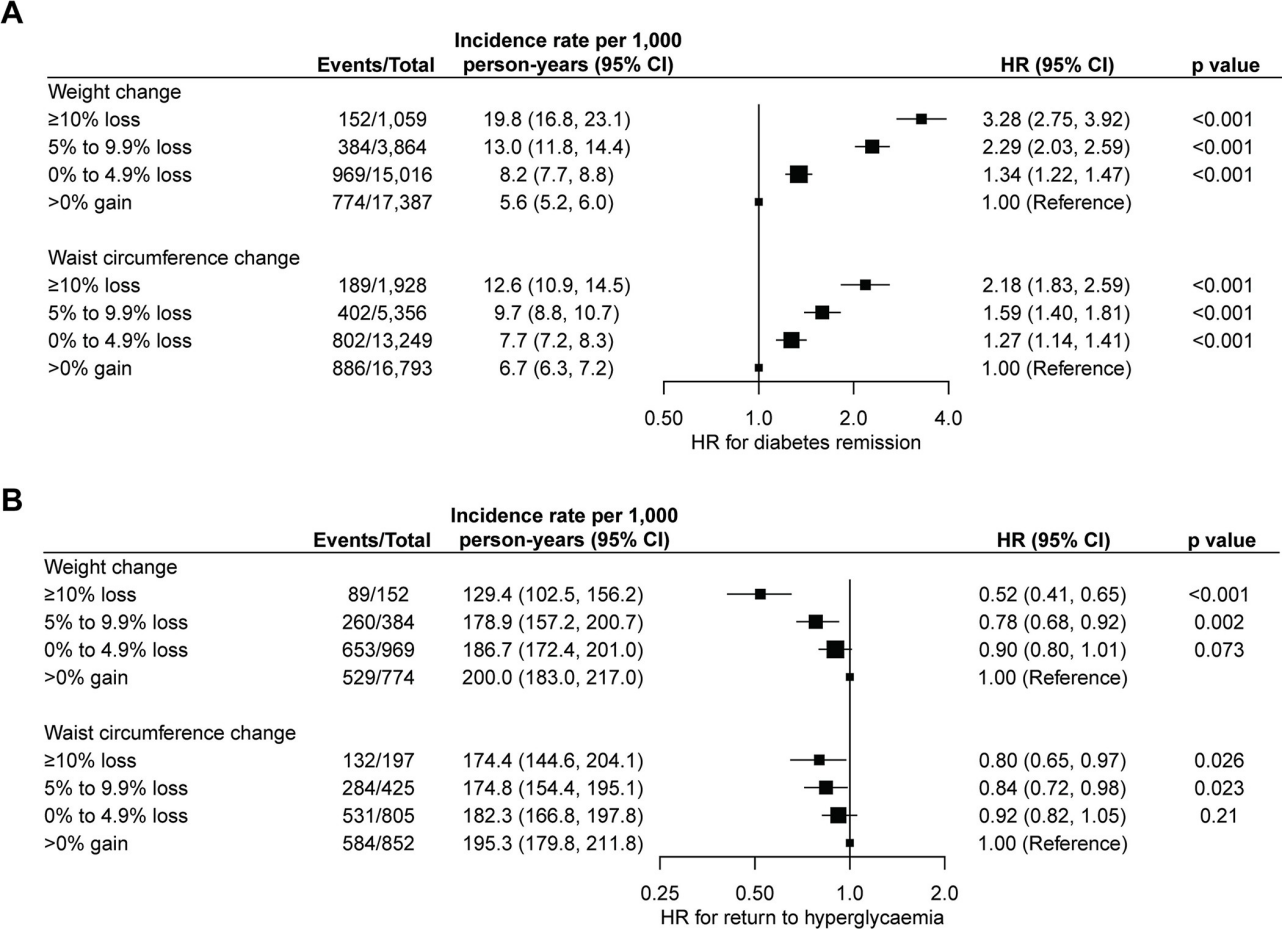
**Hazard ratios (HRs) for the associations of 1-year change (%) in weight and waist circumference after diabetes diagnosis with incident remission of type 2 diabetes (A) and incident return to hyperglycaemia among people with diabetes remission (B).** For the analyses of diabetes remission, the HRs were adjusted for age at diabetes diagnosis, sex, assessment year, BMI, waist circumference, HbA1c, SBP, LDL-C, HDL-C, triglycerides, eGFR, smoking, alcohol drinking, oral glucose-lowering drugs, blood pressure–lowering drugs, and lipid-lowering drugs. For the analyses of return to hyperglycaemia, the HRs were additionally adjusted for diabetes duration. Squares represent HRs and lines represent 95% CIs. The area of each square is inversely proportional to the variance of log HR, which also determines the 95% CI. The axis for HR is natural log-transformed. Abbreviations: BMI, body mass index; CI, confidence interval; DBP, diastolic blood pressure; eGFR, estimated glomerular filtration rate; HR, hazard ratio; HbA1c, haemoglobin A1c; HDL-C, high-density lipoprotein cholesterol; LDL-C, low-density lipoprotein; SBP, systolic blood pressure.

During a median follow-up of 7.9 years (IQR: 4.8, 10.5), 6.1% (*n* = 2,279) of people achieved diabetes remission. The overall crude incidence rate of diabetes remission was 7.8 (95% CI: 7.5, 8.1) per 1,000 person-years and 88% of the remission events occurred within the first 5 years of the follow-up (**Fig 2**). Characteristics of people with and without diabetes remission are shown in the **S2 Table**. The proportion of people achieving diabetes remission was higher among those with greater weight loss. Specifically, 14.4% (*n* = 152) of people who lost ≥10% of their body weight achieved remission, compared to 9.9% (*n* = 384) in those with a 5% to 9.9% weight loss, 6.5% (*n* = 969) in those with a 0% to 4.9% weight loss, and 4.5% (*n* = 774) in those who experienced weight gain. The restricted cubic spline model showed a nonlinear association between 1-year weight change (%) and diabetes remission (*p* < 0.001 for nonlinearity) (**Fig 3**). The HR for diabetes remission increased with increasing weight loss but was constant as the gain in weight increased. Compared to people with weight gain, the adjusted HR for diabetes remission was 3.28 (95% CI: 2.75, 3.92; *p* < 0.001) for those with ≥10% weight loss, 2.29 (95% CI: 2.03, 2.59; *p* < 0.001) for those with 5% to 9.9% weight loss, and 1.34 (95% CI: 1.22, 1.47; *p* < 0.001) for those with 0% to 4.9% weight loss (**Fig 4 and S3 Table**). In subgroup analysis, the strength of the association between 1-year weight change (%) and diabetes remission was stronger in people who had a higher HbA1c level (*p* < 0.001 for interaction) and those with central obesity (*p* = 0.003 for interaction) at baseline but did not appear to be modified by other characteristics of the study population (**S1 Fig**). The association between 1-year waist circumference change (%) and diabetes remission was similar in direction but was weaker in magnitude (for the same % change) compared to the 1-year weight change (%) (**Figs 4 and S2 and S3 Table**).

### Weight change and incident return to hyperglycaemia

During a median follow-up of 3.1 years (IQR: 2.1, 4.7) from the date of diabetes remission, 67.2% (*n* = 1,531) of people who had achieved diabetes remission returned to hyperglycaemia, with a crude incidence rate of 184.8 (95% CI: 175.5, 194.0) per 1,000 person-years. Among those who achieved diabetes remission, 99.9% (*n* = 2,277) had at least one more valid HbA1c measurement following the HbA1c measurements that confirmed their remission status during the follow-up. Around 39% of people retuned to hyperglycaemia within 3 years and 58% within 5 years after achieving remission (**Fig 2**). During the follow-up, mean body weight increased by 0.82% (SD: 5.8%) compared to the weight measured at 1 year after diabetes diagnosis in people who returned to hyperglycaemia, whereas it decreased by 0.88% (SD: 7.7%) in those who maintained remission (**S3 Fig**). The median time to return to hyperglycaemia was 3.6 (95% CI: 3.4, 3.8) years. Characteristics of people who returned and those did not return to hyperglycaemia are shown in the **S4 Table**. Greater 1-year weight loss after diabetes diagnosis was associated with a decreased risk of returning to hyperglycaemia (**Fig 4 and S5 Table**). Compared to people with weight gain, the adjusted HR for returning to hyperglycaemia was 0.52 (95% CI: 0.41, 0.65; *p* < 0.001) for those with ≥10% weight loss, 0.78 (95% CI: 0.68, 0.92; *p* = 0.002) for those with 5% to 9.9% weight loss, and 0.90 (95% CI: 0.80, 1.01; *p* = 0.073) for those with 0% to 4.9% weight loss. Similarly, the HR for returning to hyperglycaemia decreased with larger reduction in waist circumference.

### Diabetes remission and risk of mortality

**Fig 5:**
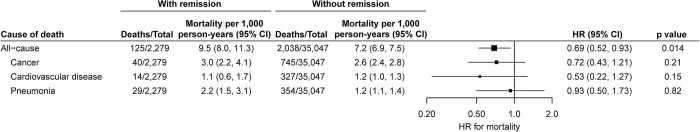
Crude mortality rates and adjusted hazard ratios (HRs) for the association of incident diabetes remission with all-cause and cause-specific mortality. The HRs were adjusted for 1-year change (%) in weight, age at diabetes diagnosis, sex, assessment year, BMI, waist circumference, HbA1c, SBP, LDL-C, HDL-C, triglycerides, eGFR, smoking, alcohol drinking, oral glucose-lowering drugs, blood pressure–lowering drugs, lipid-lowering drugs, and diabetes duration. Squares represent HRs and lines represent 95% CIs. The area of each square is inversely proportional to the variance of log HR, which also determines the 95% CI. The axis for HR is natural log-transformed. Abbreviations: BMI, body mass index; CI, confidence interval; DBP, diastolic blood pressure; eGFR, estimated glomerular filtration rate; HR, hazard ratio; HbA1c, haemoglobin A1c; HDL-C, high-density lipoprotein cholesterol; LDL-C, low-density lipoprotein; SBP, systolic blood pressure.

During a median follow-up of 7.9 years (IQR: 5.0, 10.4) for mortality, 2,163 deaths were recorded, of which 785 were due to cancer, 341 to cardiovascular disease, and 383 to pneumonia. People who experienced diabetes remission had a significantly decreased risk of all-cause mortality (HR: 0.69, 95% CI: 0.52, 0.93; *p* = 0.014) compared to those not achieving diabetes remission (**Fig 5**). On sub-analysis, the decreased risks for mortality from cancer (HR: 0.72, 95% CI: 0.43, 1.21; *p* = 0.21), cardiovascular disease (HR: 0.53, 95% CI: 0.22, 1.27; *p* = 0.15) or pneumonia (HR: 0.93, 95% CI: 0.50, 1.73; *p* = 0.82) were not statistically significant at a level of 0.05 but remained in the same direction. Among those with diabetes remission who returned to hyperglycaemia, there was a trend that the HR for all-cause mortality decreased as the duration of remission increased (*p* = 0.032 for trend) (**S4 Fig**).

### Additional and sensitivity analyses

The association between diabetes remission and all-cause mortality remained significant (HR: 0.71, 95% CI: 0.53, 0.95; *p* = 0.023) after excluding people (*n* = 34) who died within 6 months after achieving their first HbA1c measurement <6.5%. The HRs for the association between 1-year weight change (%) and diabetes remission changed little after further including people with preexisting cardiovascular disease (**S6 Table**). There were statistically significant differences in baseline characteristics between people included in the analysis and those excluded due to missing data on weight change (**S7 Table**). However, the absolute differences were small.

## Discussion

In this large long-term cohort study of Hong Kong Chinese with newly diagnosed type 2 diabetes, greater weight loss 1 year after diabetes diagnosis was associated with an increased likelihood of incident diabetes remission as well as a decreased risk of returning to hyperglycaemia among those who had achieved diabetes remission. People who experienced diabetes remission had a lower risk of all-cause mortality compared to those without diabetes remission. However, the overall incidence of diabetes remission was considerably low, with only 6% of people achieving remission over a median follow-up of 8 years. Maintaining long-term remission was challenging as approximately half of those with remission returned to hyperglycaemia within 3 years after achieving remission.

The incidence of diabetes remission in our study was comparable to that in other epidemiological studies but significantly lower than in clinical trials. A cohort study of 122,781 US adults with type 2 diabetes, in which remission was defined as 2 HbA1c measurements without use of GLD in the range of 5.7% to 6.4% over at least 12 months, reported an incidence of 2.8 per 1,000 person-years in the overall study population and 8.8 per 1,000 person-years among those with new-onset diabetes [19]. A study of 2 million people with type 2 diabetes in primary care settings in England, using the same remission definition as our study, reported an incidence of 9.7 per 1,000 person-years in the overall population and 44.9 per 1,000 person-years among people with newly diagnosed diabetes [14]. However, in the DiRECT clinical trial, 73% of participants who lost 10 kg or more body weight achieved diabetes remission (defined as an HbA1c <6.5% with no GLD for at least 2 months) at 12 months, following interventions that included total diet replacement with a low-energy formula diet, stepped food reintroduction, and structured support for long-term weight loss maintenance [3]. In the DIADEM-I clinical trial, 61% of participants in the intervention group achieved diabetes remission (defined as an HbA1c <6.5% with no GLD for at least 3 months) at 12 months following a similar regimen used in the DiRECT trial, which resulted in an average weight loss of 12 kg [4]. By contrast, our study found that only 14% of people who experienced a weight loss of 10% or more and 9.9% of those with a weight loss between 5% and 10% achieved remission over 8 years, respectively. The incidence of diabetes remission in our study was also significantly lower than that in the control groups of the DiRECT [3] and DIADEM-I [4] clinical trials, where 4% and 12% of participants achieved remission at 12 months, accompanied by average weight losses of 1 kg and 4 kg, respectively.

A number of factors might contribute to the observed discrepancy between clinical trials and studies in real-world settings. Participants in clinical trials underwent intensive lifestyle interventions that comprised complex dietary intervention, physical exercise, and cognitive-behavioural support. These structured programmes included regular monitoring, feedback, and reinforcement to provide a holistic approach to managing diabetes. However, it is unclear whether people in real-world settings engaged in any dietary modifications or other targeted interventions. Moreover, the majority of clinical trials adopted a less stringent remission criterion, generally utilising a single HbA1c measurement [3,4], in comparison to epidemiological studies that usually required 2 HbA1c measurements over time [14,16,19]. Prescription practices also influence the incidence of remission in real-world settings. Doctors may be less inclined to discontinue GLDs for their patients, even when their HbA1c level approaches normal. This practice could inadvertently limit the opportunities for patients to demonstrate potential remission. However, in both DiRECT and DIADEM-I clinical trials [3,4], all participants discontinued GLDs at the start of the intervention. More importantly, during our study period, the concept of achieving diabetes remission was not as widely recognised or incorporated into clinical practice. Innovative weight management strategies, which have been proven effective for achieving diabetes remission in recent clinical trials [3,4,20], were neither implemented nor available in real-world settings in our locality. This partially explains the low incidence of diabetes remission observed in our study, reflecting the conventional management approaches of the era.

Our study focused on weight changes during the first year following a diabetes diagnosis, a period that serves as a prime opportunity for individuals to initiate lifestyle modifications in response to their diagnosis. We found that any degree of weight loss was associated with increased likelihood of diabetes remission. People who lost ≥10% of their body weight within 1 year after diabetes diagnosis were 3 times more likely to achieve diabetes remission, and those who lost 5% to 9.9% were twice as likely, compared to those who experienced weight gain. This highlights the potential of early weight management as an important intervention to improve glycaemic control and achieve diabetes remission in real-world settings. Although our study observed a dose–response relationship between weight loss and incidence of diabetes remission, it is important to emphasise that the decision to undertake weight loss as part of diabetes management should consider priorities beyond diabetes remission and be tailored to each individual’s clinical profile. It is also noteworthy that over 80% of remission cases occurred within the first 5 years following diabetes diagnosis, and remission rarely developed in people with a long disease duration, irrespective of baseline weight changes. This is consistent with other studies suggesting that a longer duration of diabetes was associated with a reduced likelihood of remission [14,16,19], related to progressive deterioration of beta cell function under conventional management [21]. This is also supported by the Counterbalance study, which found that following adequate weight loss on a very low–calorie diet and weight maintenance intervention with concomitant cessation of GLD in people with type 2 diabetes, those who did not achieve fasting blood glucose levels <7 mmol/L (nonresponders) had a considerably greater beta cell defect and a longer diabetes duration at baseline compared to responders [22].

Our study observed a significant association between weight loss and diabetes remission even among people with a BMI <24 kg/m^2^. This finding is supported by the ReTUNE clinical trial in the United Kingdom, which showed that 70% of participants with normal or near-normal BMI (<27 kg/m^2^) went into diabetes remission through diet-induced weight loss [23]. The accumulation of intrahepatic and intrapancreatic fat is one of the key mechanisms underlying pathogenesis of type 2 diabetes, which increases hepatic insulin resistance and beta-cell function, respectively [23–25]. Weight loss could lead to reduction in these specific fat deposits irrespective of BMI, potentially contributing to diabetes remission [23].

Although type 2 diabetes is potentially reversible, remission was attained in only 6% of people over an 8-year follow-up period in our study. Notably, maintaining long-term remission proved to be a challenge. Approximately 20% of people who achieved remission returned to hyperglycaemia annually. This aligns with results from clinical trials. In the Look AHEAD study, 30% of those in the intervention group who achieved remission (defined as an HbA1c <6.5% with no GLD) returned to a clinical diabetes status every single year [26]. The DiRECT study reported that among those who achieved remission at the 12 months of intervention, 22% returned to hyperglycaemia by 24 months [3,20]. The low remission rate and the difficulty in maintaining remission reflect the complex nature of diabetes. Sustaining long-term diabetes remission may require a comprehensive and multifaceted approach that includes ongoing commitment to a healthy lifestyle and weight management with maintenance of near normoglycaemia. New gut hormone-based compounds promise large reduction in body weight in addition to blood glucose lowering [27,28]. However, clinical trials have shown rapid weight regain following withdrawal of glucagon-like peptide-1 receptor agonists [29]. The impact of diabetes remission on long-term health outcomes has not been well studied. Our study found a 30% reduction in all-cause mortality among people who experienced diabetes remission compared to those who did not. Diabetes remission, which results in a reduction of hyperglycaemia, could lower the cumulative glycaemic burden, thereby preventing diabetes-related complications and mortality [30].

This study has a number of strengths, including a large sample size, high-quality data, and a long-term follow-up period up to 20 years, which enabled us to comprehensively capture long-term patterns of diabetes remission in real-world settings. This study also has several limitations. First, HbA1c alone may not always be an appropriate test to define diabetes remission, because its reliability can be affected by other medical conditions such as anaemia and haemoglobinopathy [31]. The consensus report [13] recommends HbA1c as the usual criterion for diabetes remission due to its clinical utility, but other glucose criteria should be used if HbA1c is unavailable or deemed unreliable. Although we lacked data on anaemia and haemoglobinopathy, we used at least 2 HbA1c values to define diabetes remission. This approach could adequately identify people with sustained changes in glycaemic control and provide reliable assessment of diabetes remission. Second, we did not have data on bariatric surgery, which can lead to diabetes remission predominantly through its effects on weight loss and alterations in nutrition [24,32–34]. However, bariatric surgery is performed infrequently in Hong Kong, even among those with obesity-related complications, including diabetes [35]. Third, our study only examined weight change 1 year after diabetes diagnosis and did not capture the full spectrum of weight fluctuations over the study period. Fourth, the HA EMR system did not capture GLD prescriptions and HbA1c measurements in the private sector, which would affect the incidence of both diabetes remission and return to hyperglycaemia. However, this bias should be minimal and is likely to be nondifferential across different weight change groups. Fifth, a large proportion of people were excluded from the analysis due to missing data on weight change. This resulted in a sample that was not representative of the RAMP-DM cohort and potentially limited the generalisability of our findings. However, the absolute differences in baseline characteristics between the people included and those excluded were small.

The findings of this study demonstrate the important role of early weight management in achieving and maintaining diabetes remission in real-world settings. The low incidence of diabetes remission with conventional management and the difficulty in maintaining it underscore the importance of setting realistic expectations and prioritising the global effort of using both public and personalised measures to prevent the onset of diabetes in high-risk individuals. Innovative weight management programmes, including low-calorie diets and total meal replacement for the treatment of type 2 diabetes and obesity, have demonstrated efficacy in achieving diabetes remission in clinical trials. However, further studies are needed to examine the feasibility, sustainability, and cost-effectiveness of these innovative weight management interventions at the population level to ensure they can be applied broadly and safely to benefit a wider population in real-world settings. Moreover, the association between diabetes remission and other clinical outcomes warrants examination.

In conclusion, our findings suggest that remission of type 2 diabetes is achievable in real-world settings. Greater weight loss within the first year of diabetes diagnosis was associated with an increased likelihood of achieving diabetes remission and a decreased risk of returning to hyperglycaemia among those who had achieved diabetes remission. However, both the incidence of diabetes remission and the probability of its long-term sustainability were low with conventional management in real-world settings. Our study provides evidence for policymakers to design and implement early weight management interventions and diabetes remission initiatives.

## Supporting information

S1 STROBE Checklist STROBE Statement Checklist. (DOC)

S1 Table Proportion (%) of missing data and methods for imputation. (DOCX)

S2 Table Baseline characteristics and selected 1-year measures of the study population according to incident remission of type 2 diabetes. (DOCX)

S3 Table Hazard ratios (HRs) for the associations of 1-year change (%) in weight and waist circumference after diabetes diagnosis with incident remission of type 2 diabetes. (DOCX)

S4 Table Baseline characteristics and selected 1-year measures of people with remission of type 2 diabetes stratified by subsequent return to hyperglycaemia. (DOCX)

S5 Table Hazard ratios (HRs) for the associations of 1-year change (%) in weight and waist circumference after diabetes diagnosis with subsequent return to hyperglycaemia among people with remission of type 2 diabetes. (DOCX)

S6 Table Hazard ratios (HRs) for the associations of 1-year change (%) in weight and waist circumference after diabetes diagnosis with incident remission of type 2 diabetes in the study population (*n* = 40,282) that further included people with preexisting cardiovascular disease. (DOCX)

S7 Table Baseline characteristics of people included in the analysis compared to those excluded due to missing data on 1-year weight change but met other inclusion criteria. (DOCX)

S1 Fig Hazard ratios (HRs) for the association of 1-year change (%) in weight after diabetes diagnosis with incident remission of type 2 diabetes according to baseline characteristics. (DOCX)

S2 Fig Hazard ratios (HRs) for the association of 1-year change (%) in waist circumference after diabetes diagnosis with incident remission of type 2 diabetes according to baseline characteristics. (DOCX)

S3 Fig Proportion of people who had weight gain compared to their weight measured at 1 year after diabetes diagnosis during the follow-up for return to hyperglycaemia. (DOCX)

S4 Fig Hazard ratios for the association of remission duration with all-cause mortality in people with diabetes remission who returned to hyperglycaemia. (DOCX)

## Abbreviations

CI: confidence interval
DiRECT: Diabetes Remission Clinical Trial
EMR: Electronic Medical Record
GLD: glucose-lowering drug
HA: Hospital Authority
HbA1c: haemoglobin A1C
HR: hazard ratio
MICE: multiple imputation by chained equations
PH: proportional hazard
RAMP-DM: Risk Assessment and Management Programme for Diabetes Mellitus
SD: standard deviation
