# Supplementary material for: 1-year weight change after diabetes diagnosis and long-term incidence and sustainability of remission of type 2 diabetes in real-world settings in Hong Kong: An observational cohort study
Source: PLoS Med. 2024 Jan 23;21(1):e1004327. doi: 10.1371/journal.pmed.1004327 (PMC10805283; doi:10.1371/journal.pmed.1004327)
Supplement: S1 Table — (DOCX) [file pmed.1004327.s002.docx]

**S1 Table. Proportion (%) of missing data and methods for imputation.**

| **Variables** | **Proportion (%)** |
| --- | --- |
| Waist circumference at baseline | 11.6 |
| Waist circumference at 1 year | 2.9 |
| 1-year waist circumference change | 12.7 |
| HbA1c at baseline | 3.3 |
| HbA1c at 1 year | 1.3 |
| 1-year HbA1c change | 3.3 |
| SBP | 6.9 |
| DBP | 6.9 |
| Total cholesterol | 12.2 |
| LDL-C | 12.9 |
| HDL-C | 12.6 |
| Triglycerides | 12.2 |
| eGFR | 4.7 |
| Smoking status | 7.6 |
| Alcohol drinking status | 8.8 |

All other variables had complete data. We applied multiple imputation for Cox regression models. The variables that were included in the multiple imputation model were: age at diabetes diagnosis, sex, assessment year, BMI, 1-year weight change, HbA1c, waist circumference, 1-year waist circumference change, SBP, DBP, total cholesterol, LDL-C, HDL-C, triglycerides, eGFR, smoking, alcohol drinking, oral glucose-lowering drug, blood pressure-lowering drugs, lipid-lowering drug, incident diabetes remission, time to diabetes remission, incident death, and time to death. Imputation was performed using predictive mean matching for continuous variables, logistic regression for binary variables, and polytomous regression for categorical variables with more than two categories. We generated 15 complete imputed datasets following guidance to use at least as many imputed datasets as the percentage of observations with incomplete data. We pooled the results using Rubin’s rules.
