# Supplementary material for: 1-year weight change after diabetes diagnosis and long-term incidence and sustainability of remission of type 2 diabetes in real-world settings in Hong Kong: An observational cohort study
Source: PLoS Med. 2024 Jan 23;21(1):e1004327. doi: 10.1371/journal.pmed.1004327 (PMC10805283; doi:10.1371/journal.pmed.1004327)
Supplement: S2 Table — (DOCX) [file pmed.1004327.s003.docx]

**S2 Table.** **Baseline characteristics and selected 1-year measures of the study population according to incident remission of type 2 diabetes.**

| Characteristics | Incident remission | | p |
| --- | --- | --- | --- |
|  | Yes | No |  |
| Number (%) | 2,279 (6.1) | 35,047 (93.9) |  |
| Age at diabetes diagnosis (years) | 58.5 (10.1) | 56.5 (9.9) | <0.001 |
| Male sex | 1,061 (46.6) | 17,771 (50.7) | <0.001 |
| Assessment year |  |  | <0.001 |
| 2000-2009 | 705 (30.9) | 12,052 (34.4) |  |
| 2010-2013 | 1,074 (47.1) | 12,675 (36.2) |  |
| 2014-2017 | 500 (21.9) | 10,320 (29.4) |  |
| BMI category |  |  | 0.601 |
| <24 kg/m^2^ | 697 (30.6) | 10,421 (29.7) |  |
| 24-27.9 kg/m^2^ | 917 (40.2) | 14,106 (40.2) |  |
| ≥28 kg/m^2^ | 665 (29.2) | 10,520 (30.0) |  |
| BMI (kg/m^2^) at baseline | 26.3 (4.3) | 26.4 (4.2) | 0.673 |
| BMI (kg/m^2^) at 1 year | 25.8 (4.2) | 26.3 (4.2) | <0.001 |
| Weight (kg) at baseline | 67.0 (12.9) | 67.7 (12.9) | 0.010 |
| Weight (kg) at 1 year | 65.6 (12.7) | 67.6 (13.1) | <0.001 |
| 1-year absolute weight change (kg) | -1.4 (3.8) | -0.1 (3.5) | <0.001 |
| 1-year weight change (%) | -1.9 (5.4) | 0.0 (5.1) | <0.001 |
| Central obesity (%) | 1,362 (67.6) | 21,164 (68.3) | 0.503 |
| Waist circumference (cm) at baseline |  |  |  |
| Men | 91.2 (10.1) | 91.6 (10.1) | 0.296 |
| Women | 87.3 (10.8) | 88.0 (10.2) | 0.022 |
| Waist circumference (cm) at 1 year |  |  |  |
| Men | 89.8 (9.9) | 91.7 (10.0) | <0.001 |
| Women | 86.4 (10.5) | 87.8 (10.3) | <0.001 |
| 1-year waist circumference change (%) |  |  |  |
| Men | -1.4 (6.1) | 0.3 (5.9) | <0.001 |
| Women | -0.8 (7.3) | -0.1 (6.8) | 0.002 |
| HbA1c at baseline |  |  |  |
| % | 6.6 (1.1) | 7.7 (1.8) | <0.001 |
| mmol/mol | 48.8 (11.8) | 61.0 (20.0) | <0.001 |
| HbA1c at 1 year |  |  |  |
| % | 6.2 (0.6) | 6.9 (1.0) | <0.001 |
| mmol/mol | 44.2 (6.2) | 52.3 (11.1) | <0.001 |
| 1-year HbA1c change |  |  |  |
| % | -0.4 (1.0) | -0.8 (1.8) | <0.001 |
| mmol/mol | -4.6 (11.3) | -8.8 (19.8) | <0.001 |
| Blood pressure (mm Hg) |  |  |  |
| SBP | 135.0 (18.0) | 133.8 (17.5) | 0.003 |
| DBP | 77.0 (10.2) | 78.0 (10.2) | <0.001 |
| Total cholesterol (mmol/L) | 5.0 (0.9) | 5.0 (1.0) | 0.392 |
| LDL-C (mmol/L) | 3.0 (0.8) | 3.0 (0.9) | 0.309 |
| HDL-C (mmol/L) | 1.3 (0.3) | 1.2 (0.3) | <0.001 |
| Triglycerides (mmol/L) | 1.3 (0.9, 1.8) | 1.4 (1.0, 2.0) | <0.001 |
| eGFR (mL/min/1.73 m^2^) | 85.0 (17.5) | 90.7 (16.1) | <0.001 |
| Smoking status |  |  | <0.001 |
| Current | 186 (8.9) | 4,878 (15.0) |  |
| Former | 284 (13.6) | 4,664 (14.4) |  |
| Never | 1,611 (77.4) | 22,871 (70.6) |  |
| Alcohol drinking status |  |  | 0.016 |
| Current | 434 (21.2) | 7,399 (23.1) |  |
| Former | 142 (6.9) | 2,543 (7.9) |  |
| Never | 1,474 (71.9) | 22,048 (68.9) |  |
| Oral glucose-lowering drugs (yes) |  |  |  |
| Any | 599 (26.3) | 23,655 (67.5) | <0.001 |
| Metformin | 482 (21.1) | 20,417 (58.3) | <0.001 |
| Sulfonylureas | 188 (8.2) | 8,702 (24.8) | <0.001 |
| Others | 2 (0.1) | 87 (0.2) | 0.193 |
| Blood pressure-lowering drugs (yes) | 1,548 (67.9) | 18,501 (52.8) | <0.001 |
| Lipid-lowering drugs (yes) | 408 (17.9) | 6,817 (19.5) | 0.074 |

Data are mean (standard deviation), median (interquartile range), or n (%) as appropriate. Summary statistics are reported based on the complete data for each variable. Central obesity is defined as waist circumference ≥90 cm in men and waist circumference ≥80 cm in women. Abbreviations: BMI, body mass index; DBP, Diastolic blood pressure; eGFR, estimated glomerular filtration rate, HbA1c, haemoglobin A1c; HDL-C, high-density lipoprotein cholesterol; LDL-C, low-density lipoprotein; SBP, systolic blood pressure.
