# Supplementary material for: 1-year weight change after diabetes diagnosis and long-term incidence and sustainability of remission of type 2 diabetes in real-world settings in Hong Kong: An observational cohort study
Source: PLoS Med. 2024 Jan 23;21(1):e1004327. doi: 10.1371/journal.pmed.1004327 (PMC10805283; doi:10.1371/journal.pmed.1004327)
Supplement: S3 Table — (DOCX) [file pmed.1004327.s004.docx]

**S3 Table.** **Hazard ratios (HRs) for the associations of 1-year change (%) in weight and waist circumference after diabetes diagnosis with incident remission of type 2 diabetes.**

| Study group | Model 1 | | Model 2 | | Model 3 | | Model 4 | |
| --- | --- | --- | --- | --- | --- | --- | --- | --- |
|  | HR (95% CI) | p | HR (95% CI) | p | HR (95% CI) | p | HR (95% CI) | p |
| Overall |  |  |  |  |  |  |  |  |
| Weight change |  |  |  |  |  |  |  |  |
| ≥10% loss | 3.41 (2.87, 4.01) | <0.001 | 3.28 (2.76, 3.91) | <0.001 | 3.76 (3.16, 4.48) | <0.001 | 3.28 (2.75, 3.92) | <0.001 |
| 5% to 9.9% loss | 2.29 (2.03, 2.59) | <0.001 | 2.21 (1.95, 2.50) | <0.001 | 2.45 (2.16, 2.77) | <0.001 | 2.29 (2.03, 2.59) | <0.001 |
| 0% to 4.9% loss | 1.46 (1.33, 1.61) | <0.001 | 1.42 (1.29, 1.56) | <0.001 | 1.45 (1.31, 1.59) | <0.001 | 1.34 (1.22, 1.47) | <0.001 |
| >0% gain | 1.0 (Reference) |  | 1.0 (Reference) |  | 1.0 (Reference) |  | 1.0 (Reference) |  |
| Waist circumference change |  |  |  |  |  |  |  |  |
| ≥10% loss | 2.02 (1.72, 2.39) | <0.001 | 1.87 (1.59, 2.19) | <0.001 | 2.09 (1.78, 2.46) | <0.001 | 2.18 (1.83, 2.59) | <0.001 |
| 5% to 9.9% loss | 1.51 (1.33, 1.71) | <0.001 | 1.45 (1.30, 1.64) | <0.001 | 1.57 (1.39, 1.78) | <0.001 | 1.59 (1.40, 1.81) | <0.001 |
| 0% to 4.9% loss | 1.20 (1.09, 1.33) | <0.001 | 1,18 (1.06, 1.31) | 0.002 | 1.23 (1.11, 1.37) | <0.001 | 1.27 (1.14, 1.41) | <0.001 |
| >0% gain | 1.0 (Reference) |  | 1.0 (Reference) |  | 1.0 (Reference) |  | 1.0 (Reference) |  |

Model 1: unadjusted model.

Model 2: adjusted for age at diabetes diagnosis, sex, and assessment year.

Model 3: additionally adjusted for baseline BMI (or waist circumference for 1-year waist circumference change) and HbA1c based on Model 2.

Model 4: additionally adjusted for baseline waist circumference (or BMI for 1-year weight change), SBP, LDL-C, HDL-C, triglycerides, eGFR, smoking, alcohol drinking, oral glucose-lowering drugs, blood pressure-lowering drugs, and lipid-lowering drugs based on Model 3.

Abbreviations: BMI, body mass index; DBP, Diastolic blood pressure; eGFR, estimated glomerular filtration rate, HbA1c, haemoglobin A1c; HDL-C, high-density lipoprotein cholesterol; LDL-C, low-density lipoprotein; SBP, systolic blood pressure.
