# Supplementary material for: 1-year weight change after diabetes diagnosis and long-term incidence and sustainability of remission of type 2 diabetes in real-world settings in Hong Kong: An observational cohort study
Source: PLoS Med. 2024 Jan 23;21(1):e1004327. doi: 10.1371/journal.pmed.1004327 (PMC10805283; doi:10.1371/journal.pmed.1004327)
Supplement: S4 Table — (DOCX) [file pmed.1004327.s005.docx]

**S4 Table. Baseline characteristics and selected 1-year measures of people with remission of type 2 diabetes stratified by subsequent return to hyperglycaemia.**

| Characteristics | Returned to hyperglycarmia | | p |
| --- | --- | --- | --- |
|  | Yes | No |  |
| Number (%) | 1,531 (67.2) | 748 (32.8) |  |
| Age at diabetes diagnosis (years) | 57.8 (10.0) | 60.0 (10.2) | <0.001 |
| Male sex | 687 (44.9) | 374 (50.0) | 0.024 |
| Assessment year |  |  | <0.001 |
| 2000-2009 | 540 (35.3) | 165 (22.1) |  |
| 2010-2013 | 738 (48.2) | 336 (44.9) |  |
| 2014-2017 | 253 (16.5) | 247 (33.0) |  |
| BMI category |  |  | 0.151 |
| <24 kg/m^2^ | 452 (29.5) | 245 (32.8) |  |
| 24-27.9 kg/m^2^ | 636 (41.5) | 281 (37.6) |  |
| ≥28 kg/m^2^ | 443 (28.9) | 222 (29.7) |  |
| BMI (kg/m^2^) at baseline | 26.3 (4.0) | 26.4 (4.7) | 0.591 |
| BMI (kg/m^2^) at 1 year | 25.8 (4.0) | 25.8 (4.4) | 0.820 |
| Weight (kg) at baseline | 66.8 (12.5) | 67.2 (13.8) | 0.522 |
| Weight (kg) at 1 year | 65.6 (12.4) | 65.6 (13.3) | 0.956 |
| 1-year absolute weight change (kg) | -1.3 (3.5) | -1.6 (4.2) | 0.044 |
| 1-year weight change (%) | -1.8 (5.1) | -2.2 (5.8) | 0.110 |
| Central obesity (%) | 926 (68.3) | 436 (66.0) | 0.308 |
| Waist circumference (cm) at baseline |  |  |  |
| Men | 91.1 (9.5) | 91.4 (11.0) | 0.713 |
| Women | 86.8 (10.4) | 88.4 (11.6) | 0.021 |
| Waist circumference (cm) at 1 year |  |  |  |
| Men | 89.8 (9.6) | 89.8 (10.4) | 0.980 |
| Women | 86.1 (10.3) | 87.1 (10.8) | 0.133 |
| 1-year waist circumference change (%) |  |  |  |
| Men | -1.4 (5.9) | -1.3 (6.4) | 0.862 |
| Women | -0.6 (7.4) | -1.2 (7.2) | 0.189 |
| HbA1c at baseline |  |  |  |
| % | 6.6 (1.0) | 6.5 (1.2) | 0.015 |
| mmol/mol | 49.3 (10.9) | 48.0 (13.4) | 0.015 |
| HbA1c at 1 year |  |  |  |
| % | 6.2 (0.5) | 6.1 (0.7) | <0.001 |
| mmol/mol | 44.8 (5.1) | 43.0 (8.0) | <0.001 |
| 1-year HbA1c change |  |  |  |
| % | -0.4 (1.0) | -0.5 (1.1) | 0.319 |
| mmol/mol | -4.5 (10.9) | -5.0 (12.1) | 0.319 |
| Blood pressure (mm Hg) |  |  |  |
| SBP | 135.1 (18.1) | 134.6 (17.8) | 0.569 |
| DBP | 77.3 (10.1) | 76.4 (10.3) | 0.061 |
| Total cholesterol (mmol/L) | 5.0 (0.9) | 4.9 (1.0) | <0.001 |
| LDL-C (mmol/L) | 3.0 (0.8) | 2.9 (0.8) | 0.001 |
| HDL-C (mmol/L) | 1.3 (0.3) | 1.3 (0.3) | 0.543 |
| Triglycerides (mmol/L) | 1.3 (0.9, 1.8) | 1.2 (0.9, 1.7) | 0.023 |
| eGFR (mL/min/1.73 m^2^) | 85.6 (17.5) | 83.9 (17.6) | 0.038 |
| Smoking status |  |  | 0.915 |
| Current | 123 (8.8) | 63 (9.3) |  |
| Former | 190 (13.6) | 94 (13.8) |  |
| Never | 1,088 (77.7) | 523 (76.9) |  |
| Alcohol drinking status |  |  | 0.290 |
| Current | 279 (20.2) | 155 (23.1) |  |
| Former | 99 (7.2) | 43 (6.4) |  |
| Never | 1,002 (72.6) | 472 (70.4) |  |
| Oral glucose-lowering drugs (yes) |  |  |  |
| Any | 343 (22.4) | 256 (34.2) | <0.001 |
| Metformin | 266 (17.4) | 216 (28.9) | <0.001 |
| Sulfonylureas | 123 (8.0) | 65 (8.7) | 0.650 |
| Others | 1 (0.1) | 1 (0.1) | 1 |
| Blood pressure-lowering drugs (yes) | 1,030 (67.3) | 518 (69.3) | 0.368 |
| Lipid-lowering drugs (yes) | 247 (16.1) | 161 (21.5) | 0.002 |

Data are mean (standard deviation), median (interquartile range), or n (%) as appropriate. Summary statistics are reported based on the complete data for each variable. Central obesity is defined as waist circumference ≥90 cm in men and waist circumference ≥80 cm in women. Abbreviations: BMI, body mass index; DBP, Diastolic blood pressure; eGFR, estimated glomerular filtration rate, HbA1c, haemoglobin A1c; HDL-C, high-density lipoprotein cholesterol; LDL-C, low-density lipoprotein; SBP, systolic blood pressure.
