# Supplementary material for: 1-year weight change after diabetes diagnosis and long-term incidence and sustainability of remission of type 2 diabetes in real-world settings in Hong Kong: An observational cohort study
Source: PLoS Med. 2024 Jan 23;21(1):e1004327. doi: 10.1371/journal.pmed.1004327 (PMC10805283; doi:10.1371/journal.pmed.1004327)
Supplement: S5 Table — (DOCX) [file pmed.1004327.s006.docx]

**S5 Table.** **Hazard ratios (HRs) for the associations of 1-year change (%) in weight and waist circumference after diabetes diagnosis with** **subsequent return to hyperglycaemia among people with remission of type 2 diabetes.**

| Study group | Model 1 | | Model 2 | | Model 3 | | Model 4 | |
| --- | --- | --- | --- | --- | --- | --- | --- | --- |
|  | HR (95% CI) | p | HR (95% CI) | p | HR (95% CI) | p | HR (95% CI) | p |
| Overall |  |  |  |  |  |  |  |  |
| Weight change |  |  |  |  |  |  |  |  |
| ≥10% loss | 0.57 (0.46, 0.72) | <0.001 | 0.55 (0.44, 0.69) | <0.001 | 0.51 (0.41, 0.65) | <0.001 | 0.52 (0.41, 0.65) | <0.001 |
| 5% to 9.9% loss | 0.85 (0.73, 0.99) | 0.036 | 0.85 (0.73, 0.98) | 0.030 | 0.82 (0.70, 0.95) | 0.009 | 0.78 (0.68, 0.92) | 0.002 |
| 0% to 4.9% loss | 0.91 (0.81, 1.02) | 0.10 | 0.93 (0.83, 1.04) | 0.22 | 0.93 (0.83, 1.04) | 0.20 | 0.90 (0.80, 1.01) | 0.073 |
| >0% gain | 1.0 (Reference) |  | 1.0 (Reference) |  | 1.0 (Reference) |  | 1.0 (Reference) |  |
| Waist circumference change |  |  |  |  |  |  |  |  |
| ≥10% loss | 0.84 (0.69, 1.03) | 0.098 | 0.85 (0.70, 1.03) | 0.090 | 0.82 (0.68, 0.99) | 0.045 | 0.80 (0.65, 0.97) | 0.026 |
| 5% to 9.9% loss | 0.85 (0.73, 0.99) | 0.039 | 0.86 (0.74, 0.99) | 0.036 | 0.85 (0.73, 0.98) | 0.022 | 0.84 (0.72, 0.98) | 0.023 |
| 0% to 4.9% loss | 0.95 (0.84, 1.08) | 0.44 | 0.95 (0.84, 1.07) | 0.39 | 0.94 (0.84, 1.06) | 0.34 | 0.92 (0.82, 1.05) | 0.21 |
| >0% gain | 1.0 (Reference) |  | 1.0 (Reference) |  | 1.0 (Reference) |  | 1.0 (Reference) |  |

Model 1: unadjusted model.

Model 2: adjusted for age at diabetes diagnosis, sex, assessment year, and diabetes duration at the time of remission.

Model 3: additionally adjusted for baseline BMI (or waist circumference for 1-year waist circumference change) and HbA1c based on Model 2.

Model 4: additionally adjusted for baseline waist circumference (or BMI for 1-year weight change), SBP, LDL-C, HDL-C, triglycerides, eGFR, smoking, alcohol drinking, oral glucose-lowering drugs, blood pressure-lowering drugs, and lipid-lowering drugs based on Model 3.

Abbreviations: BMI, body mass index; DBP, Diastolic blood pressure; eGFR, estimated glomerular filtration rate, HbA1c, haemoglobin A1c; HDL-C, high-density lipoprotein cholesterol; LDL-C, low-density lipoprotein; SBP, systolic blood pressure.
