# Supplementary material for: 1-year weight change after diabetes diagnosis and long-term incidence and sustainability of remission of type 2 diabetes in real-world settings in Hong Kong: An observational cohort study
Source: PLoS Med. 2024 Jan 23;21(1):e1004327. doi: 10.1371/journal.pmed.1004327 (PMC10805283; doi:10.1371/journal.pmed.1004327)
Supplement: S6 Table — (DOCX) [file pmed.1004327.s007.docx]

**S6 Table.** **Hazard ratios (HRs) for the associations of 1-year change (%) in weight and waist circumference after diabetes diagnosis with incident remission of type 2 diabetes in the study population (n=40,282) that further included people with pre-existing cardiovascular disease.**

| Study group | Model 1 | | Model 2 | | Model 3 | | Model 4 | |
| --- | --- | --- | --- | --- | --- | --- | --- | --- |
|  | HR (95% CI) | p | HR (95% CI) | p | HR (95% CI) | p | HR (95% CI) | p |
| Overall |  |  |  |  |  |  |  |  |
| Weight change |  |  |  |  |  |  |  |  |
| ≥10% loss | 3.44 (2.89, 4.09) | <0.001 | 3.30 (2.77, 3.93) | <0.001 | 3.80 (3.19, 4.53) | <0.001 | 3.31 (2.78, 3.94) | <0.001 |
| 5% to 9.9% loss | 2.30 (2.04, 2.60) | <0.001 | 2.22 (1.96, 2.51) | <0.001 | 2.47 (2.18, 2.79) | <0.001 | 2.27 (2.00, 2.57) | <0.001 |
| 0% to 4.9% loss | 1.46 (1.33, 1.61) | <0.001 | 1.42 (1.29, 1.56) | <0.001 | 1.46 (1.33, 1.60) | <0.001 | 1.34 (1.22, 1.47) | <0.001 |
| >0% gain | 1.0 (Reference) |  | 1.0 (Reference) |  | 1.0 (Reference) |  | 1.0 (Reference) |  |
| Waist circumference change |  |  |  |  |  |  |  |  |
| ≥10% loss | 2.06 (1.74, 2.42) | <0.001 | 1.92 (1.62, 2.27) | <0.001 | 2.12 (1.79, 2.52) | <0.001 | 2.20 (1.84, 2.63) | <0.001 |
| 5% to 9.9% loss | 1.52 (1.34, 1.72) | <0.001 | 1.45 (1.29, 1.65) | <0.001 | 1.58 (1.39, 1.80) | <0.001 | 1.58 (1.38, 1.80) | <0.001 |
| 0% to 4.9% loss | 1.20 (1.08, 1.33) | <0.001 | 1.18 (1.06, 1.30) | 0.002 | 1.24 (1.11, 1.37) | <0.001 | 1.26 (1.14, 1.40) | <0.001 |
| >0% gain | 1.0 (Reference) |  | 1.0 (Reference) |  | 1.0 (Reference) |  | 1.0 (Reference) |  |

Model 1: unadjusted model.

Model 2: adjusted for age at diabetes diagnosis, sex, and assessment year.

Model 3: additionally adjusted for baseline BMI (or waist circumference for 1-year waist circumference change) and HbA1c based on Model 2.

Model 4: additionally adjusted for baseline waist circumference (or BMI for 1-year weight change), SBP, LDL-C, HDL-C, triglycerides, eGFR, smoking, alcohol drinking, oral glucose-lowering drugs, blood pressure-lowering drugs, lipid-lowering drugs, and history of cardiovascular disease based on Model 3.

Abbreviations: BMI, body mass index; DBP, Diastolic blood pressure; eGFR, estimated glomerular filtration rate, HbA1c, haemoglobin A1c; HDL-C, high-density lipoprotein cholesterol; LDL-C, low-density lipoprotein; SBP, systolic blood pressure.
