# Supplementary material for: 1-year weight change after diabetes diagnosis and long-term incidence and sustainability of remission of type 2 diabetes in real-world settings in Hong Kong: An observational cohort study
Source: PLoS Med. 2024 Jan 23;21(1):e1004327. doi: 10.1371/journal.pmed.1004327 (PMC10805283; doi:10.1371/journal.pmed.1004327)
Supplement: S7 Table — (DOCX) [file pmed.1004327.s008.docx]

**S7 Table. Baseline characteristics of people included in the analysis compared to those excluded due to missing data on 1-year weight change but met other inclusion criteria.**

| Characteristics | Population | | p |
| --- | --- | --- | --- |
|  | Included in the analysis | Excluded from the analysis |  |
| Number | 37,326 | 117,328 |  |
| Age at diabetes diagnosis (years) | 56.6 (9.9) | 56.6 (10.0) | 0.55 |
| Male sex | 18,832 (50.5) | 60,236 (51.3) | 0.003 |
| Assessment year |  |  | <0.001 |
| 2000-2009 | 12,757 (34.2) | 20,666 (17.6) |  |
| 2010-2013 | 13,749 (36.8) | 38,747 (33.0) |  |
| 2014-2017 | 10,820 (29.0) | 57,915 (49.4) |  |
| BMI category |  |  | <0.001 |
| <24 kg/m^2^ | 11,118 (29.8) | 30,119 (29.2) |  |
| 24-27.9 kg/m^2^ | 15,023 (40.2) | 40,584 (39.4) |  |
| ≥28 kg/m^2^ | 11,185 (30.0) | 32,336 (31.4) |  |
| BMI (kg/m^2^) | 26.4 (4.2) | 26.5 (4.3) | <0.001 |
| Weight (kg) | 67.6 (12.9) | 68.6 (13.7) | <0.001 |
| Central obesity (%) | 22,526 (68.3) | 70,817 (70.4) | <0.001 |
| Waist circumference (cm) at baseline |  |  |  |
| Men | 91.5 (10.2) | 92.4 (10.4) | <0.001 |
| Women | 87.9 (10.3) | 88.8 (10.4) | <0.001 |
| HbA1c at baseline |  |  |  |
| % | 7.7 (1.8) | 7.5 (1.7) | <0.001 |
| mmol/mol | 60.3 (19.9) | 58.9 (18.8) | <0.001 |
| Blood pressure (mm Hg) |  |  |  |
| SBP | 133.8 (17.5) | 132.5 (16.9) | <0.001 |
| DBP | 78.0 (10.2) | 77.6 (10.3) | <0.001 |
| Total cholesterol (mmol/L) | 5.0 (1.0) | 4.9 (1.0) | <0.001 |
| LDL-C (mmol/L) | 3.0 (0.9) | 2.9 (0.9) | <0.001 |
| HDL-C (mmol/L) | 1.3 (0.3) | 1.3 (0.3) | 0.008 |
| Triglycerides (mmol/L) | 1.4 (1.0, 2.0) | 1.4 (1.0, 2.0) | <0.001 |
| eGFR (mL/min/1.73 m^2^) | 90.3 (16.2) | 90.3 (16.3) | 0.84 |
| Smoking status |  |  | <0.001 |
| Current | 5,064 (14.7) | 17,123 (16.4) |  |
| Former | 4,948 (14.3) | 14,985 (14.4) |  |
| Never | 24,482 (71.0) | 71,985 (69.2) |  |
| Alcohol drinking status |  |  | <0.001 |
| Current | 7,833 (23.0) | 24,658 (24.0) |  |
| Former | 2,685 (7.9) | 7,420 (7.2) |  |
| Never | 23,522 (69.1) | 70,489 (68.7) |  |
| Oral glucose-lowering drugs (yes) |  |  |  |
| Any | 24,254 (65.0) | 76,599 (65.3) | 0.28 |
| Metformin | 20,899 (56.0) | 69,116 (58.9) | <0.001 |
| Sulfonylureas | 8,890 (23.8) | 25,041 (21.3) | <0.001 |
| Others | 89 (0.2) | 540 (0.5) | <0.001 |
| Blood pressure-lowering drugs (yes) | 20,049 (53.7) | 66,100 (56.3) | <0.001 |
| Lipid-lowering drugs (yes) | 7,225 (19.4) | 35,535 (30.3) | <0.001 |

Data are mean (standard deviation), median (interquartile range), or n (%) as appropriate. Summary statistics are reported based on the complete data for each variable. Central obesity is defined as waist circumference ≥90 cm in men and waist circumference ≥80 cm in women. Abbreviations: BMI, body mass index; DBP, Diastolic blood pressure; eGFR, estimated glomerular filtration rate, HbA1c, haemoglobin A1c; HDL-C, high-density lipoprotein cholesterol; LDL-C, low-density lipoprotein; SBP, systolic blood pressure.
