# Supplementary material for: 1-year weight change after diabetes diagnosis and long-term incidence and sustainability of remission of type 2 diabetes in real-world settings in Hong Kong: An observational cohort study
Source: PLoS Med. 2024 Jan 23;21(1):e1004327. doi: 10.1371/journal.pmed.1004327 (PMC10805283; doi:10.1371/journal.pmed.1004327)

**S2 Fig. Hazard ratios (HRs) for the association of 1-year change (%) in waist circumference after diabetes diagnosis with incident remission of type 2 diabetes according to baseline characteristics.** The adjusted hazard ratios were stratified by (A) age at diabetes diagnosis (p=0.13 for interaction), (B) sex (p=0.083 for interaction), (C) baseline HbA1c (p<0.013 for interaction), (D) general obesity (p=0.79 for interaction), (E) central obesity (p=0.40 for interaction), and (F) baseline eGFR (p=0.93 for interaction). The adjusted hazard ratios were based on the fully adjusted model. Central obesity is defined as waist circumference ≥90 cm in men and waist circumference ≥80 cm in women. Dots represent HRs and lines represent 95% CIs. Abbreviations: CI, confidence interval; eGFR, estimated glomerular filtration rate.


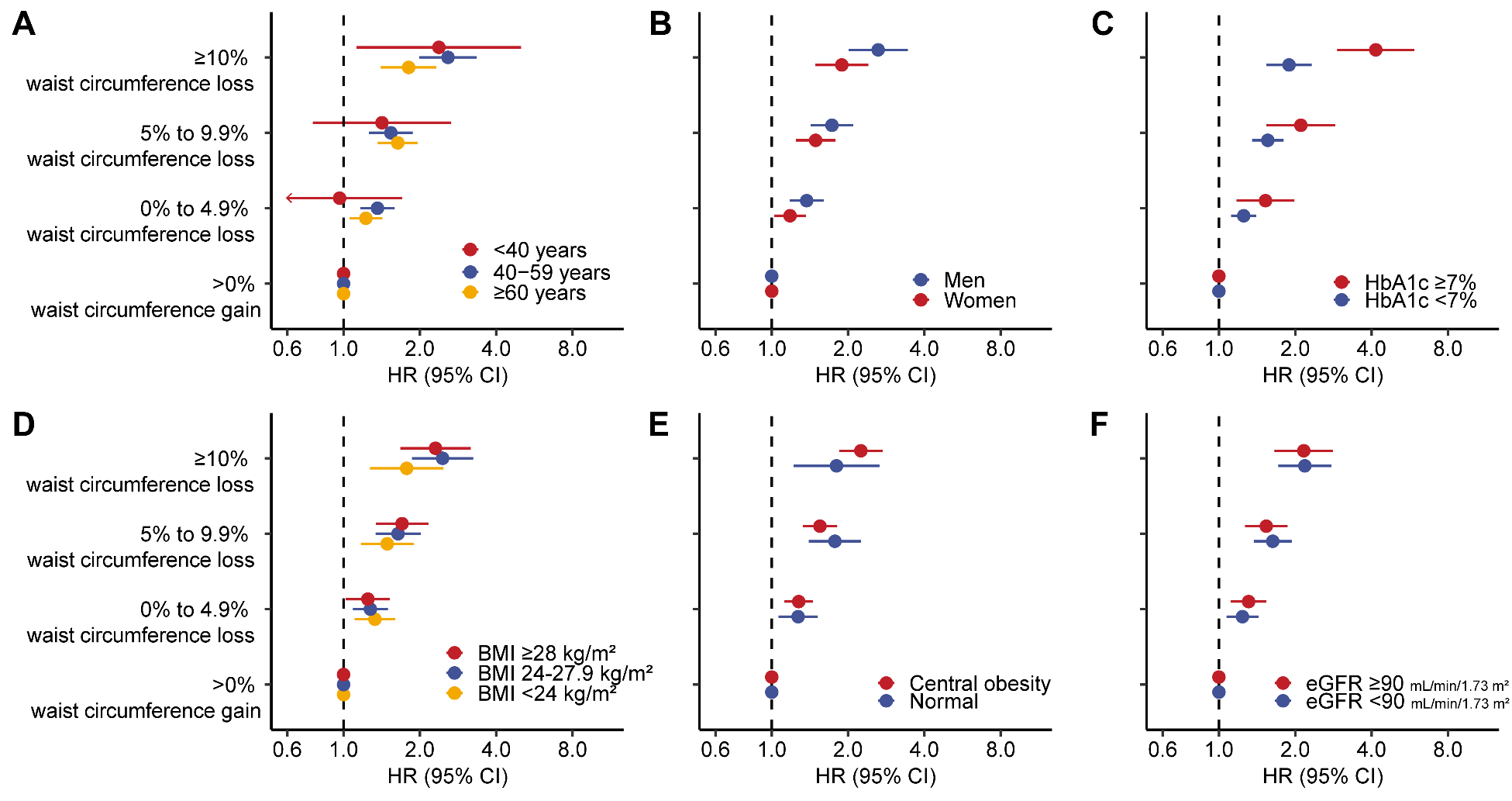

Supplement: S2 Fig — (DOCX) [file pmed.1004327.s010.docx]
