# Supplementary material for: 1-year weight change after diabetes diagnosis and long-term incidence and sustainability of remission of type 2 diabetes in real-world settings in Hong Kong: An observational cohort study
Source: PLoS Med. 2024 Jan 23;21(1):e1004327. doi: 10.1371/journal.pmed.1004327 (PMC10805283; doi:10.1371/journal.pmed.1004327)
Supplement: S3 Fig — (DOCX) [file pmed.1004327.s011.docx]

**S3 Fig.** **Proportion of people who had weight gain compared to their weight measured at 1 year after diabetes diagnosis during the follow-up for return to hyperglycaemia.** The proportion was calculated based on 1,788 (out of 2,279) people with diabetes remission who had available weight records during the follow-up for return to hyperglycaemia. For people who had multiple weight records during the follow-up, the last record was used. Bars represent the proportion and lines represent the 95% confidence intervals.

**
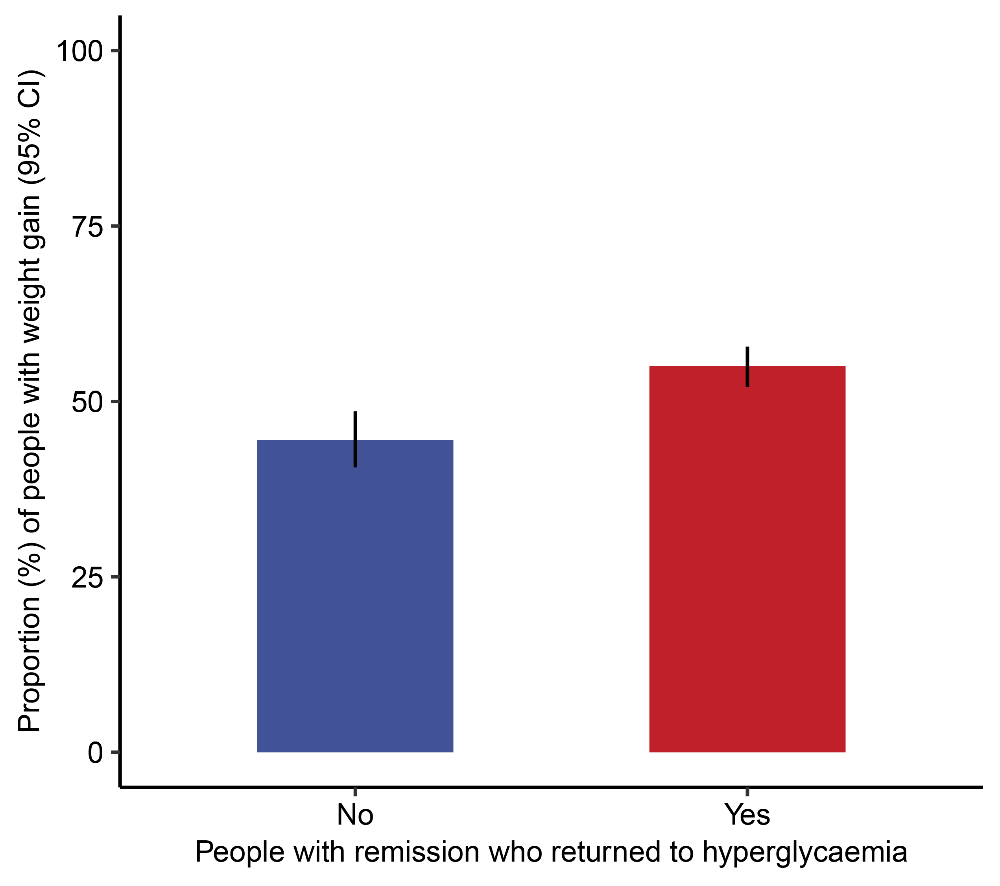
**
