# Supplementary material for: 1-year weight change after diabetes diagnosis and long-term incidence and sustainability of remission of type 2 diabetes in real-world settings in Hong Kong: An observational cohort study
Source: PLoS Med. 2024 Jan 23;21(1):e1004327. doi: 10.1371/journal.pmed.1004327 (PMC10805283; doi:10.1371/journal.pmed.1004327)

**S4 Fig. Hazard ratios for the association of remission duration with all-cause mortality in people with diabetes remission who returned to hyperglycaemia.** The HRs were adjusted for 1-year change (%) in weight, age at diabetes diagnosis, sex, assessment year, BMI, waist circumference, HbA1c, SBP, LDL-C, HDL-C, triglycerides, eGFR, smoking, alcohol drinking, oral glucose-lowering drugs, blood pressure-lowering drugs, lipid-lowering drugs, and diabetes duration. All people were followed up from the date of return to hyperglycaemia to avoid immortal time bias. Squares represent HRs and lines represent 95% CIs. The area of each square is inversely proportional to the variance of log HR, which also determines the 95% CI. The axis for HR is natural log-transformed. Abbreviations: BMI, body mass index; CI, confidence interval; DBP, Diastolic blood pressure; eGFR, estimated glomerular filtration rate; HR: hazard ratio; HbA1c, haemoglobin A1c; HDL-C, high-density lipoprotein cholesterol; LDL-C, low-density lipoprotein; SBP, systolic blood pressure.


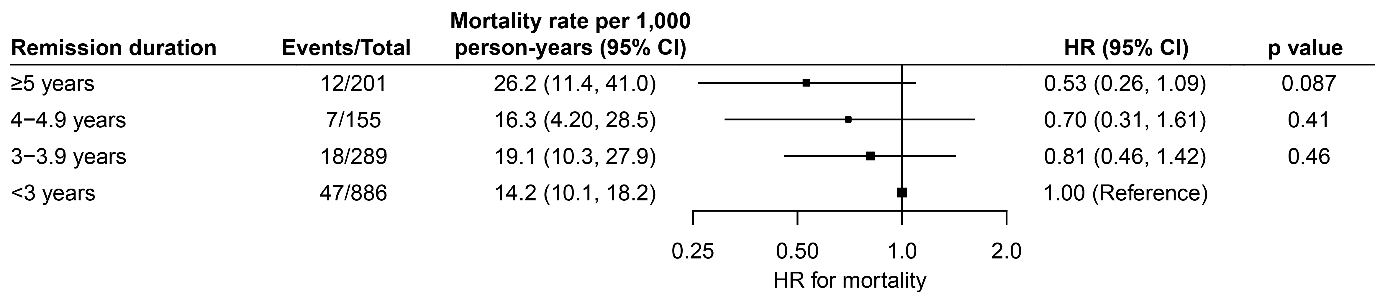

Supplement: S4 Fig — (DOCX) [file pmed.1004327.s012.docx]
